# Consequences of COVID-19 on adolescents in Arizona: A longitudinal study protocol

**DOI:** 10.3389/fpubh.2022.945089

**Published:** 2022-12-15

**Authors:** Velia Leybas Nuño, Namoonga M. Mantina, Oriyomi Dawodu, Maureen Dykinga, Dametreea L. Carr, Kristen Pogreba-Brown, Felina Cordova-Marks, Megan Jehn, Kimberly Peace-Tuskey, Leila Barraza, Pamela Garcia-Filion

**Affiliations:** ^1^Department of Health Promotion Sciences, Mel and Enid Zuckerman College of Public Health (MEZCOPH), University of Arizona, Tucson, AZ, United States; ^2^Department of Health Promotion Sciences, MEZCOPH, University of Arizona, Tucson, AZ, United States; ^3^Department of Epidemiology and Biostatistics, MEZCOPH, University of Arizona, Tucson, AZ, United States; ^4^School of Human Evolution and Social Change, Arizona States University, Tempe, AZ, United States; ^5^Department of Community, Environment and Policy, MEZCOPH, University of Arizona, Tucson, AZ, United States; ^6^College of Medicine, University of Arizona, Phoenix, AZ, United States

**Keywords:** COVID-19, adolescents, depressive symptoms, anxiety, resilience, epidemiology, intersectionality

## Abstract

**Introduction:**

The long-term impact of COVID-19 is unknown. We developed a 5-year prospective cohort study designed to generate actionable community-informed research about the consequences of COVID-19 on adolescents ages 12–17 years in Arizona.

**Methods:**

The study has two primary outcomes: 1) acute and long-term outcomes of COVID-19 illness and 2) symptoms of depression and anxiety. Data is collected using an online survey with plans to integrate qualitative data collection methods. The survey is administered at baseline, 4, and 8 months in year one, and annually in years two through five. This study is informed by Intersectionality Theory, which considers the diverse identities adolescents have that are self and socially defined and the influence they have collectively and simultaneously. To this end, a sample of variables collected is race/ethnicity, language usage, generational status, co-occurring health conditions, and gender. Additional measures capture experiences in social contexts such as home (parent employment, food, and housing security), school (remote learning, type of school), and society (racism).

**Results:**

Findings are not presented because the manuscript is a protocol designed to describe the procedure instead of report results.

**Discussion:**

The unique contributions of the study is its focus on COVID-19 the illness and COVID-19 the socially experienced pandemic and the impact of both on adolescents.

## Introduction

COVID-19 has greatly influenced the lives of adolescents in direct and indirect ways. The extent of influence is unknown, therefore longitudinal studies are imperative. Globally 464 million people and over 78 million people in the United States (U.S.) have contracted SARS-CoV-2, the virus that causes COVID-19 (1). Among the 73 million U.S. children and adolescents, 17,095 per 100,000 have had COVID-19 (2). Cases resulting in the hospitalization of children and adolescents are 1–5% and deaths < 0.02% (2). In Arizona, over two million of the state's 7,303,398 population have been infected with SARS-CoV-2 (3, 4). Twenty-one percent of the cumulative cases in the state are 19 years of age and younger, and 65 deaths (3). To prevent severe illness, hospitalizations, and death, efficacious vaccinations are available. Seventeen million or 68% of adolescents 12–17 years of age in the U.S. have had at least one dose of the COVID-19 vaccine (2). Fourteen and a half million or 58% have received two doses (2). Among Arizona adolescents 19 years of age and younger, 36% have had at least one dose (3).

### Description of Arizona

Arizona is a state in the southwestern U.S. It shares state borders with Utah to the north, California and Nevada to the west, New Mexico to the east, and an international border with Mexico to the south. The international border with Mexico offers an exchange of people, culture, and goods that create unique border communities. Socially constructed classifications (5) of ethnicity and race of the Arizona population are as follows: Latinx 31%, German 17%, Irish 11%, and English 10% (6). Racial groups are Whites 77%, from another race not listed here 7%, Black or African American 5%, Native American 5%, people belonging to two or more races 4%, Asian 3%, and Native Hawaiian or Pacific Islanders < 1% (6). There are 22 federally recognized Native American tribes in the state (7). Arizona has a diverse population, and expansive open spaces. By area Arizona is the sixth largest state in the U.S. (4). Pockets of rural communities are spread across the 15-county state (8). The large distances between rural and some tribal and some border communities create a challenge to access necessities such as food and healthcare. These challenges were worsened with COVID-19 by deepening economic and health inequities.

In January of 2021 people categorized as Native Americans comprised 5–9% of the Arizona population but made up 12% of COVID-19 deaths (9). Moreover, the Navajo people experienced more COVID-19 cases per capital compared to any state in the U.S. in the spring of 2020 (10). Seventy-five percent of Navajo people reside in Arizona with the Navajo Nation spanning Arizona, New Mexico, and Utah (11). The Tohono O'odham Nation had 1,748 COVID-19 cases and 69 deaths in March of 2021 for a population of 33,000 enrolled members (12). Health inequities are also among people classified as Latinx or Black. Nationally, those categorized as Latinx had 1.5 times more cases, 2.3 times more hospitalizations, and 1.8 times more deaths than those categorized as White (13). People categorized as Black had 1.1 more cases, 2.4 times more hospitalizations, and 1.7 times more deaths than those categorized as White (13). Life expectancy decreased early in the pandemic from 2019 to the first six months of 2020, however people classified as Black and Latinx experienced disproportionately greater loss such that Blacks had a 2 year reduction (74–72 years), Latinx a 1.9 year reduction (81.8–79.9 years), and Whites a 0.8 reduction (78.8 to 78 years) (14).

### Co-morbidities

Co-occurring and/or pre-existing conditions have increased the risk of SARS-CoV-2 infection and severity of the illness. Such a pattern is seen among children and adolescents with disabilities. More severe cases of COVID-19 have been found among children and adolescents compared to adults (15). Children and adolescents 17 years of age and younger with intellectual and developmental disabilities (IDD) had a case-fatality of 1.6% in comparison to children without IDD with < 0.01% (15). The nature of IDD can interfere with understanding and implementation of COVID-19 mitigation strategies. For some adolescents with disabilities, the disability does not appear to increase the risk of infection, but rather the changes in service provision during the pandemic placed barriers to health system's access (16).

### Inequities in basic needs

Economics contribute to the challenges posed by COVID-19. In 2020, the national poverty rate was 11% (17) and unemployment was 15% (18) still lower than that of many racial and ethnic groups in Arizona prior to the pandemic. Limited access to basic needs such as food and housing creates cascading results for adolescent development and health. In the Adolescent Behaviors and Experiences Survey conducted by the Centers for Disease Control and Prevention (CDC) from June to January 2021, 33% of high school students classified as Black reported food insecurity in their home (19). Prior to the pandemic, almost one in ten youth classified as Latinx resided in crowded housing (20). Crowding increased the risk of infection from SARS-CoV-2 among youth living in immigrant and mixed-nativity households (20). A study of 16,651 U.S. adults found 24% experienced housing inaccessibility defined as insufficient funds for rent, mortgage, or utilities or moving in with others (21). Furthermore, of these adults 77% reported worries about insufficient food, running out of food, or reducing serving sizes or skipping meals altogether. Inaccessible housing was related to no usual source of care (AOR 1.31, 95% CI 1.08–1.59), delaying care (AOR 1.84, 95% CI 1.46–2.31), and delaying medication acquisition (AOR 2.16, 95% CI 1.70–2.74) (21). Adult people of color that identified as lesbian, gay, bisexual, or transgender (LGBT; 17%) were three times more likely to experience food insecurity than White non-LGBT adults (6%) (22). The impact of stress on adolescents surrounding basic needs can be seen in findings from a national survey where 52% reported worries about family's health, 40% about finances, 39% about education, and 30% about food, medication, and safety (23). Worries impact mental health. Shortly after the pandemic began, a study found that more than 25% of high school students in the U.S. reported emotional distress manifesting in the inability to sleep, feelings of unhappiness and constant strain, and a decrease in self-confidence (23).

### Racism

Co-occurring with the pandemic, has been social injustice and striking political controversy. The events and undercurrent at points in the pandemic identify long-held racial tension that permeates the experiences of adolescents in the U.S. According to the Adolescent Behaviors and Experiences Survey, over 33% of high school adolescents reported bad or unfair treatment at school that they attributed to their race or ethnic category (19). The Weathering Hypothesis describes the negative health consequences of chronic discrimination and stress (24). Students reporting high levels of racism were in the Asian, Black, and Multiracial classifications (19). Racism negatively impacted adolescent mental health and fostered disconnectedness with school. This is particularly problematic when considering school is an important social context for adolescents to develop both socially and academically (19).

### Development

Socialization has been interrupted by prolonged separation due to school closures and social distancing measures. Peer interaction is a necessary part of development particularly during adolescence as youth move toward becoming adults. As part of that development, important structural and functional changes occur in the brain (25). Research among adolescent mice suggest social isolation has negative implications on brain processes and behavior (26). The influence of the pandemic whether directly or indirectly will have implications on adolescent development and health.

### Youth that identify as transgender

According to an analysis of the national Youth Risk Behavior Surveys from 2017 to 2019 by the Williams Institute, 1.4% or 300,000 youth in the U.S. ages 13–17 years identified as transgender (22). In Arizona, 1.54% or 7,300 youth ages 13–17 years identify as transgender. The age group of 13–17 years is the second largest group of people that identify as transgender in Arizona. The first is young adults ages18–24 years with 1.92% or 13,000 (22). The southwestern states of California (1.93% or 49,100), Texas (1.42% or 29,800), and New Mexico (2.62% or 3,700) show youth ages 13–17 years are the largest group that identifies as transgender in their states. The data suggest the younger age groups in these U.S./Mexico border states have the greater percentages of transgender identifying people. The finding is informative in recording the changing gender identification landscape or potentially reflective of a growing acceptance to identifying as transgender. These data have implications for preparing to meet the needs of youth.

### Resilience

Among the experiences during the pandemic, resilience is an important aspect. In a study published in 2021 of children and adolescents (*n* = 2,863) in Hong Kong ages nine to 17 years where 73% were categorized in the medium affluence group, youth reported greater awareness of health including noting signs of stress and responding by relaxing (27). In the U.S., adolescent resilience during the pandemic has yet to be quantified with validated survey tools. In a study of youth from Australia, utilizing the Connor-Davidson Resilience-10 (CD-RISC-10) survey, those between the ages of 12–18 years were found to have an average CD-RISC-10 score of 20.93 on a 0–40 scale (higher scores correlate to higher self-perceived resilience) (28). In a study of adolescents from China, resilience was found to be predictive of stress, anxiety, depression and post-traumatic stress disorder (29).

### Intersectionality theory

Given the many factors involved in the impact of COVID-19 on adolescents, Intersectionality Theory has been selected as a guiding theoretical approach for the design and analysis of the study. Crenshaw is credited for introducing intersectionality theory with work dating back to the 1980s (30, 31). Intersectionality Theory focuses on the multiple interdependent categories of social groups, rather than on a singular identity (32–34). It acknowledges the ways in which age, class, sexuality, gender, disability, race, ethnicity, and other social categories become mutually constructed through powerful and often limiting systems (33). The authors acknowledge that race is a classification system. It includes societal beliefs and practices that are intricately woven into the order and operation of society that perpetuates advantage and disadvantage among groups (5). An intersectional approach focuses on the impact of social, economic, and demographic characteristics and how it shapes adolescents' daily experiences and health outcomes (34).

The study collects surveys over 5 years among youth ages 12–17 years living, working, or attending school in Arizona. The objective of the study aligns with principle three of the Principles of Action from the World Health Organization's Commission on the Social Determinants of Health (35). Principle three calls for data to be collected to assess the problem. To that end, the purpose of the study is to (1) calculate the frequency of COVID-19 over time, (2) calculate the prevalence of symptoms of depression and anxiety over time, (3) create groups using PROGRESS-Plus (36) designed to measure inequities, (4) evaluate the interaction between the groups and COVID-19, depressive symptoms, and anxiety symptoms.

## Materials and methods

### Design

The CoVHORT Children and Teens Study (CATS) is a 5-year prospective cohort study. It has been developed alongside Arizona CoVHORT, an adult longitudinal study. Details about the Arizona CoVHORT protocol are in the February 10, 2021, issue of this journal (37). Adolescents eligible to participate in CATS live, work, or go to school in Arizona and are 12–17 years of age and read and write in English or Spanish. CATS is approved by the University of Arizona Human Subjects Protection Program (IRB number 2103560999) and has a Certificate of Confidentiality issued by the National Institutes of Health CC-OD-21-1467.

### Investigative team

The investigators form an interdisciplinary team that features knowledge and skills in epidemiology, social work, maternal and child health (includes adolescent health and children and youth with disabilities), health behavior health promotion, data and statistical software, speech language pathology, policy, and education. Members include students, staff, and faculty. Students are undergraduates and graduate students. Staff are skilled in epidemiology. Faculty members are assistant or associate professors. Members of the team are from the University of Arizona and Arizona State University. Fifty-six percent of the team is Indigenous, Black, and/or Latinx. Ninety-three percent are women with 50% women of color and one man of color. Twenty-five percent are bilingual (English/Spanish).

### Recruitment

Adolescents are recruited through partnerships, networks, outreach, and community engagement. The goal is to recruit 500 youth. Our partnership with the Arizona CoVHORT will provide the opportunity for ongoing recruitment. Our partnership with Arizona State University will focus recruitment efforts in Maricopa county in central Arizona. The study will distribute English and Spanish electronic and print flyers. Digital promotion of the study includes using various social media platforms and the Arizona CoVHORT/CATS website. Photos will display adolescents from intersecting identities and categories. Language will be adapted for the intended audience including parents or guardians, adolescents, or service providers. Consideration for gender-inclusive language in Spanish has been implemented with the flyers. Spanish language defers to the masculine form of words such as chicos (males) and chicas (females) therefore in the absence of a single word to include all genders (such as they), we will use chicos/as.

Through the investigative teams' networks, flyers will be sent. Furthermore, targeted recruitment at youth sporting events and local street fairs are planned. Outreach to schools is planned as is partnerships with local coalitions and county health departments. Community engagement in CATS is intended to be responsive and respectful and mutually beneficial. CATS has been building a relationship with the border community of San Luis, Arizona. Discussions involve implementing qualitative research methods that will engage undergraduate students in the training of high school youth in the research method, PhotoVoice, whereby data (photographs) are created by the youth and contextualized through narrative (38).

At the time of this writing, we were not actively recruiting adolescents from tribal communities, although the youth that are members of tribes or identify as Native American/American Indian/Indigenous may self-select to participate. Engagement with tribal communities is underway through consultation with tribal councils which must approve and/or require a memorandum of agreement before recruitment can begin. The process of approval is customized to each tribe.

### Consent and assent

**Figure 1:**
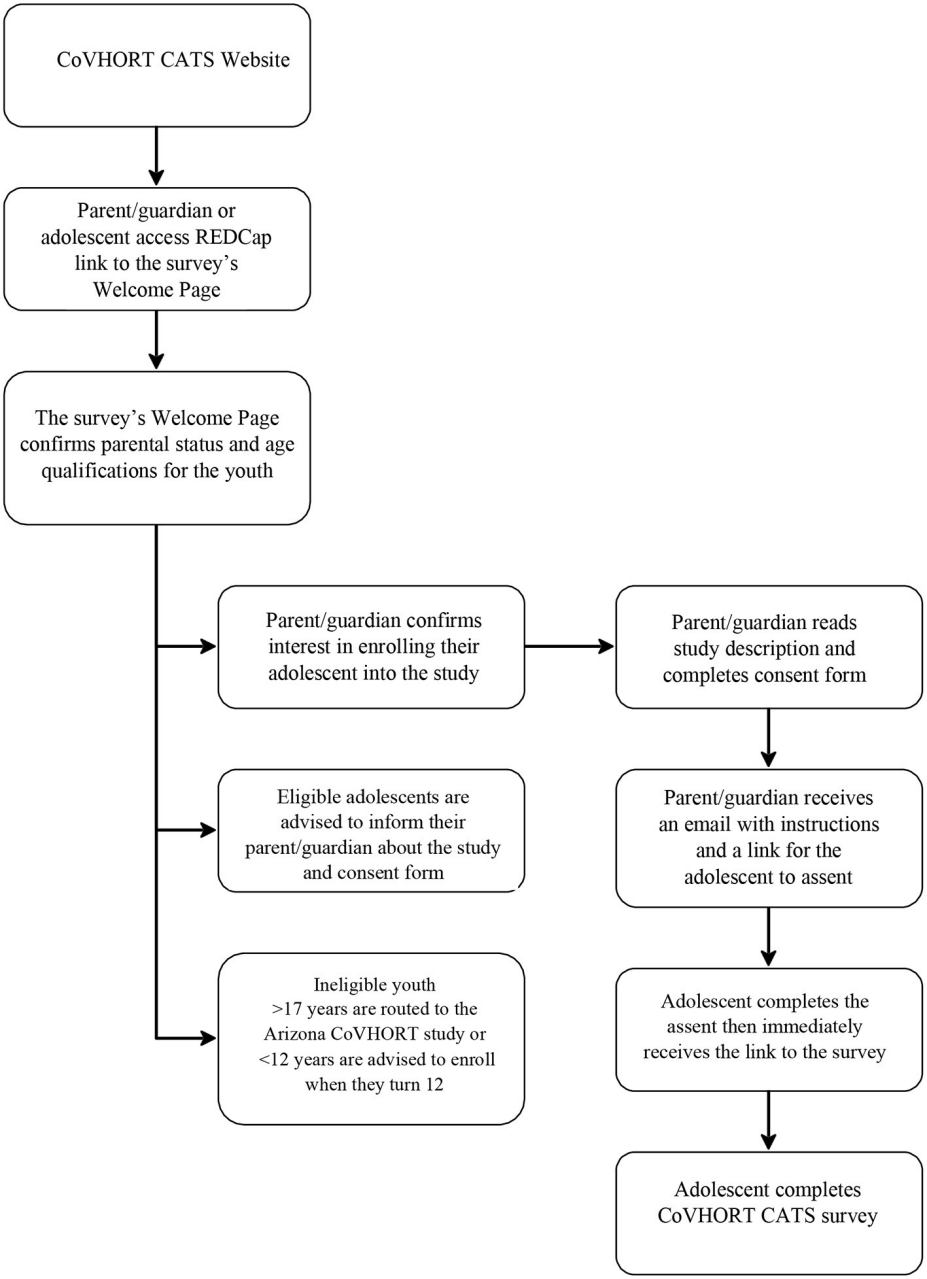
CoVHORT CATS procedural flow chart.

Access to study information is at the Arizona CoVHORT study website in the “Teens” tab (www.covhort.arizona.edu/CATS). Figure 1 shows the enrollment and survey process. The welcome page provides information about the survey, eligibility criteria, and a series of questions to confirm eligibility. The website can be translated in seconds to multiple languages using Google Translate (39). Guests are first asked if they are a parent or guardian. If they are not, then they are presumed to be adolescents and are informed to secure consent to advance to enrollment. If they are a parent or guardian, then they are guided through an electronic consent process that requires a digital signature. Up to six adolescents can be enrolled per parent or guardian. For each of the adolescents consented, parents or guardians select the preferred communication contact (parent/guardian or adolescent). This person receives study reminders and survey links. For adolescents with parent or guardian communication selected, the parent or guardian is sent an email containing a custom link to their adolescent's assent and survey. If more than one adolescent was enrolled and parent or guardian communication was selected, the parent or guardian receives instructions to have each adolescent complete the assent and baseline survey from the same device and in the same sitting. When one adolescent completes the assent and baseline survey, the parent or guardian is prompted for the next adolescent to “complete assent and survey again”. For adolescents with youth communication selection, the adolescent is emailed a personalized link to complete the assent form and baseline survey.

Adolescents have 2 weeks to complete the assent and baseline survey. The assent has been developed from evidence-based methods of language analysis in the field of speech-language pathology (40). The assent is an interactive process whereby adolescents read content in sections then answer a brief question with multiple choice options. They receive immediate feedback on their response with praise for the desired response, and a reminder of the material for the undesired response. By adding the questions, the authors intend to promote comprehension in efforts to achieve informed consent. Immediately after assent, a link is sent to the baseline survey.

### Data collection

A total of seven surveys are administered over 5 years. In year one, three surveys are collected: baseline, 4, and 8-month. In years two through five, four surveys are collected: 12, 24, 48, and 60-month. Annual surveys are administered on the anniversary of the baseline survey.

#### Data management

Consents, assents, and the survey data are collected using the Research Electronic Data Capture (REDCap), a secure, HIPAA-compliant, web-based electronic platform that supports data capture and management in research (41). Data is stored in REDCap and de-identified data from REDCap is exported to Stata 17 for analysis (42).

#### Participation

**Figure 2:**
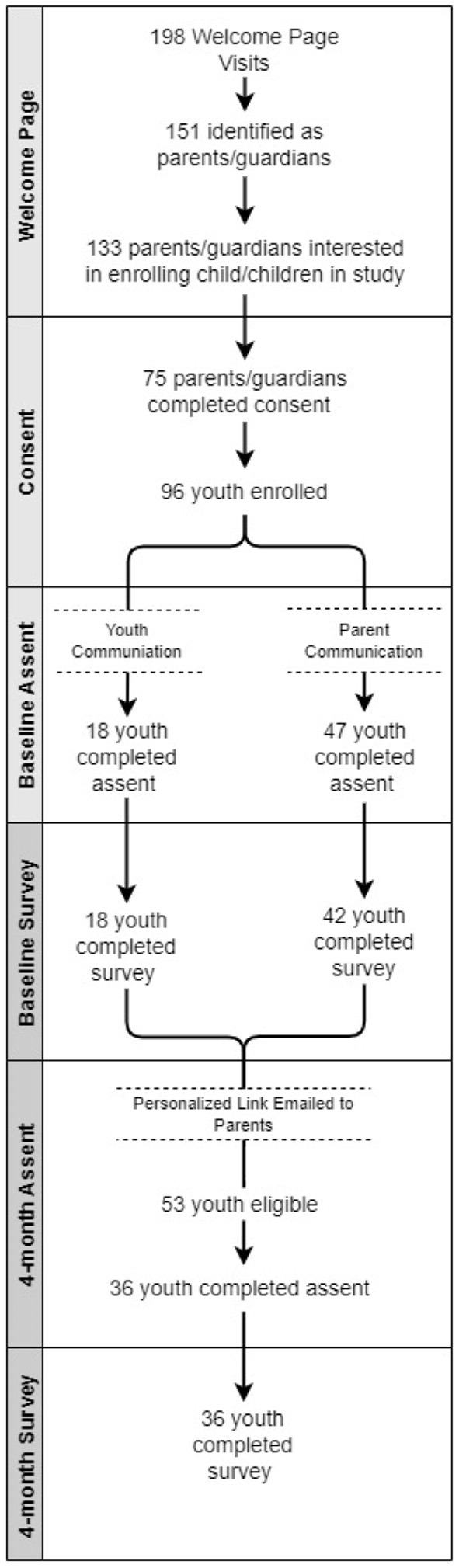
CoVHORT CATS assent and completed surveys.

The CoVHORT CATS baseline survey was officially launched July 8, 2021. Figure 2 illustrates engagement from the launch date to May 6, 2022 (302 days of the study). There have been 198 visits to the study welcome page with 133 parents or guardians expressing interest in enrolling their adolescent. At the consent stage, 75 parents or guardians have consented to enrolling their adolescent and collectively, 96 adolescents are enrolled in the study. The adolescent communication format yielded 18 adolescents who completed the assent and baseline survey. The parent or guardian communication format has yielded 47 completed assents and 42 completed surveys. In total, there are 65 completed assents and 60 completed baseline surveys as of May 6, 2022. In this timeframe 53 adolescents have been eligible for their 4-month survey; of these, 36 completed the 4-month assent and 36 completed the 4-month survey.

### Measures

The baseline survey has 79 items. The follow-up surveys measure change over time. They include the baseline questions and exclude some fixed variables such place of birth. Some measures were developed based on the expertise of the investigative team (Table 1) and some from existing, validated scales. The primary outcome variables are COVID-19, depressive symptoms, and anxiety symptoms. Variables that contribute to forming groups reflecting intersecting identities and factors include gender, sexual orientation, resilience, food and housing accessibility, race and ethnicity, racism, disability, age, place of birth, and generational status.

**Table 1:** Investigative team developed survey items for the CoVHORT Children and Teens Study, participants 12–17 years of age.

**Domains**	**Example questions**
Experiences with COVID-19	
Symptoms	What, if any, symptoms did you experience?
Evaluation and Testing	Which of the following occurred as a result of your symptoms?
	Did you (or your parent/guardian) contact a physician, urgent care, or an emergency department for this illness?
Disease Progression	Did you ever start to feel better and then get worse during the course of your illness?
Household Transmission	Has anyone else living in your home had or probably had COVID-19?
Vaccination	
Vaccine Perception	How safe do you think the COVID-19 vaccine is?
Vaccine Receipt	Have you received a COVID-19 vaccine?
School	
School Type	What kind of school do you go to?
School Format	When you were last in school were you in person, hybrid, fully online?
Online School	What do you like about going to school online?
	What do you NOT like about school online?
Home and Family	
Financial	Are you or your family worried about having enough money?
Food	How worried are you about having enough food?
Housing	How worried are you about losing your home?

The survey has one item for each of the following, race or ethnicity classification, gender identity, and sexual orientation. One item asks the adolescents about their place of birth. Six items ask about the type of school the adolescent attends and their experience with hybrid, online, and in-person modalities (Table 1).

#### COVID-19

Thirteen items ask about the adolescent's COVID-19 experience. The items are sequenced to start with whether an adolescent had COVID-19 or had been told they had COVID-19. Subsequent questions skip to items about testing, symptoms, treatment, and recovery based on previous responses. Testing questions ask if the adolescent has been tested, and if so, how such as nasal swab or blood withdrawal. Next, adolescents select all symptoms they have experienced from a list. Adolescents are then prompted to select the actions they or their caregiver took to relieve their symptoms. To capture perceived severity, adolescents are asked how sick they felt based on a scale from 0 to 10 with 0 defined as “you did not feel sick at all” and 10 as “you felt very sick”. Next, adolescents indicating their illness had passed are asked if they feel as well as they did before they were sick. Finally, adolescents are asked whether anyone else in their home had had COVID-19.

#### Vaccination

Two items ask about vaccination. One question measures perceived safety and the other measures vaccination status. The safety question is “How safe do you think the COVID-19 vaccine is?” Response options are “extremely, very, somewhat, not too much, not at all.” The vaccination status question is “Have you received a COVID-19 vaccine?” Response options are “yes, no, I don't know.”

#### Co-occurring conditions

Pre-existing conditions can increase the risk of COVID-19. Moreover, mental health conditions could have contributed to greater difficulty in school during stay-at-home orders. One item asks participants to select from 36 conditions commonly reported in the National Survey of Children with Special Health Care Needs (43). The first option is “none” followed by the 36 conditions. The item ends with the option to select “other” and then type in the condition.

#### Racism

The experience of racism can have several negative effects on the health and well-being of adolescents (44). Eleven items address racism. Two were created by the investigative team, and nine are a subset from the Adolescent Discrimination Distress Index (ADDI) (45). The ADDI measures institutional, educational, and peer discrimination experiences. Adolescents indicate if they have had a particular experience or not.

#### Home and family

The first set of items captures basic needs such as having sufficient income, food, and housing. Questions include “Are you or your family worried about having enough money?”, “How worried are you about having enough food?”, “During this past school year, did you go to school for free breakfast or lunch?”, “How worried are you about losing your home?”, and “How many times in the last year have you moved?”.

The next set of items ask about parent and grandparent nativity, language spoken at home, and interpretation. Close to 13% of people living in Arizona are foreign-born, with the majority from Latin America (6). Compared to other states, Arizona has the most people that speak an Indigenous language (6). Similarly, Arizona has a significant population that speaks Spanish. Among all ages, 20% of the Arizona population speaks Spanish and 24% of those 5–17 years of age speak Spanish (6). A meaningful percent of people immigrate to the U.S. with close to 13% of people born in countries other than the U.S., the majority are from Latin America (6).

Adolescents are asked “Were any of your biological parents or grandparents born in another country?” If yes, then the adolescent selects which family member(s) are foreign-born parents and/or grandparents. Adolescents are asked “What language or languages do you hear at home?” Following adolescents are asked “Have you translated/interpreted for your parents?” and “I have to help my parents by explaining how to do things in the US.” Finally, adolescents are asked “Have you worried about family members having trouble with immigration (for example, getting deported, getting a green card, or getting arrested)?

#### Depressive symptoms

There are 27 questions to measure mental health including depression and anxiety symptoms. Depressive symptoms are measured using the Center for Epidemiological Studies Depression Scale for Children (CES-DC), a 20-item self-report inventory that asks adolescents how they felt in the past week (46). Response options are presented using a 4-point Likert scale with four of the 20 items reverse coded. Scores range from 0 to 60 with a score of 15 suggesting depressive symptoms and scores >15 indicating severe depressive symptoms (46, 47). Symptom scores can also be categorized into a group of four ranging from mild to severe (47). Studies show the CES-DC internal consistency ranges from Cronbach's α = 0.71–0.91 (48–50) and test-retest reliability among adolescents ages 12–18 years to range from 0.70 to 0.85 (48, 50). The CES-DC has been tested and validated in other countries including India (51), Iran (49), Rwanda (52), Germany (48), and China (50).

#### Anxiety symptoms

Seven items measure anxiety using the Generalized Anxiety Disorder (GAD-7) scale (53). Scores range from 0 to 21 with severity cut-points at 5 for mild, 10 for moderate, and 15 for severe (54). A single cut-point of 8 is recommended and suggests the likelihood of anxiety (54, 55). Two studies, one with adolescents from Finland and the other with adolescents from China, together covered the ages of 10–18 years and found an internal consistency range from Cronbach's α = 0.91–0.95 (56, 57). In a separate study of adolescents in Ghana the internal consistency was Cronbach's α = 0.69 (58). The GAD-7 correlated with other scales measuring depression and mental health (58). Cultural considerations of the GAD-7 are the potential to underestimate anxiety in young adults categorized as Black/African American using the existing cut-points. In a study of undergraduates living in the US categorized as Black/African American, investigators found potential measurement bias that could result in lower anxiety scores rather than an accurate estimate of the severity of the anxiety experienced (59). The GAD-7 has been tested and validated with U.S. adolescents and adolescents from other countries such as China (57), Ghana (58), and Finland (56).

#### Resilience

Resilience, the ability of individuals to maintain well-being through the identification and acquisition of psychological, cultural, physical, and social resources, is an important measure for this study (60, 61). The Child and Youth Resilience Measure (CYRM-R) is a self-report scale designed to assess available resources that may improve resilience in youth ages 10 to 23 years (62). The internal consistency reliability in the CYRM-R is α = 0.82, reporting a good fit to the Rasch model as it is unidimensional, has good fit statistics and a lack of bias and problematic local dependency (63). Additionally, the CYRM-R has shown concurrent validity with positive correlations with self-esteem (*r* = 0.22–0.53), peer support (*r* = 0.53), and social skills (*r* = 0.62) (64). The CATS survey includes 11 items addressing resilience and is scored using a 3-point Likert scale. Adolescents answer “no” or “sometimes” or “yes” for each item.

### Statistical approach

The data will be analyzed for the primary outcomes of COVID-19, depressive symptoms, and anxiety symptoms. The rates of new (incident) and existing (prevalent) cases of COVID-19 infection will be calculated. Incident cases will be defined as new infection or meeting criteria for depression and anxiety since the baseline survey. The prevalence of COVID-19, depression, and anxiety will be based on each time point. Descriptive statistics will be calculated for all variables. Frequencies will be used for categorical data and means (± standard deviation) for continuous data. For skewed continuous data we will use the median and range. Changes over time will be captured using analyses for repeated measures such as general linear modeling.

Data analysis will be informed by Intersectionality Theory. Although there is interest in the application of Intersectionality Theory best practices for research, best practices are in development (65). We will form groups to measure inequities among outcomes using PROGRESS-Plus (36). PROGRESS-Plus has categorized individual, social, relational, and time-specific categories that have shown a relationship with inequities in health. Each letter in PROGRESS represents a category such as “S” that is for “social capital”. The “Plus” portion is for experiences of discrimination (i.e., mental or physical dis/ability), features of relationships (i.e., foreign-born grandparents), and relationships that are related to a particular time period (i.e., transition from middle to high school). Once groups are formed, we can conduct bivariate analyses using the chi-square test to compare categorical data, two-sample *t*-test to compare continuous data between groups, and the correlation coefficient to compare continuous data. Non-parametric analog methods will be used when univariate data is non-parametric. After, we can conduct regression models with the groups as interaction terms. We cannot pre-specify all the statistical tests to be performed as we cannot predict the final sample size, although we are projecting enrollment to total 500 youth. The number of groups that will be created are to be determined; however, we will follow best practices in our analytical approaches and data reporting, including pre-specified analysis plans, statistically defensible methods for missing data, thoughtful sensitivity analyses, and the careful use of reporting guidelines. Data will be analyzed in alignment with Intersectionality Theory with consideration for the multiple factors and/or identities that can work simultaneously to influence health inequities (57). Multiple imputation will be used to account for missing data if appropriate, with imputation models that include variables associated with missingness. Other sensitivity analyses may center around changing definitions of cases and/or symptoms.

## Results

Because the manuscript is a protocol paper, results are not presented, however, given the data suggest greater mental health needs among youth, we speculate that depressive symptoms and anxiety symptoms will be higher than estimates prior to the pandemic. We anticipate learning more about long COVID-19 symptoms and recovery as the research develops and through our data collection. We predict some challenges in forming intersectionality groups and draw from the collective knowledge within our investigative team, others, and the literature to inform our decision-making process.

## Discussion

The research protocol describes a study for adolescents in Arizona to measure the frequency of COVID-19, depressive symptoms, and anxiety symptoms. Our study is unique with the application of Intersectionality Theory to research with adolescents from a southwestern state along the U.S.-Mexico border. Another study in Arizona is the AZ HEROES Kids Study (66). It investigates the risk of infection from SARS-CoV-2 and vaccine effectiveness among children ages 4 months to 17 years. CATS is different from AZ HEROES Kids Study with our focus on COVID-19 and mental health. Nationally, studies from the CDC have examined the impact of COVID-19 and mental health with the CovEx survey among ages 13–19 years (67) and a cross-sectional study of high school students in 2021 to evaluate youth's behaviors and experiences of the pandemic (68). Globally, a systematic review identified 13 studies about the mental health impact of COVID-19 on children and adolescents (69). Of these one was conducted in each country including the U.S., Italy, India, and Canada, and nine in China. We have only begun to gather data on the impact of COVID-19 on the physical and mental health of adolescents. Our study can contribute to the practice, scientific, and policy communities in a few ways. Findings can inform interventions and contribute knowledge to inform future Healthy People objectives and Sustainable Development Goals. Results of the study may inform needed policies to address macro level factors that are creating systemic and structural barriers that contribute to inequities in health for youth.

While cases of COVID-19 have subsided, there is a need to understand the progression of long COVID-19. A systematic review and meta-analysis found a prevalence of 25% of long COVID-19 among children and adolescents (70). In some cases, children develop multisystem inflammatory syndrome (MIS-C). At present we are aware of these two continuing conditions from COVID-19, but it is too early to tell if other conditions will surface. In addition to the physical health implications of COVID-19, the elevated level of psychological distress is repeatedly stated in the literature. Warnings from global and national sources emphasize the critical need already apparent. In a national study of over 7,000 students in grades nine to 12 conducted in 2021, 37% reported negative mental health during the pandemic (71). In the year prior, 44% said they felt sad and hopeless.

The study has limitations. One is recall bias introduced by the survey format of the study. Additionally, some recruitment methods, such as recruiting from the Arizona CoVHORT study may introduce selection bias. Another limitation is recruitment. Great effort is required to reach adolescents from across the state. It requires partnerships across Arizona to engage youth, and partnerships take time to cultivate. Furthermore, partnerships should be mutually beneficial. The process of partnering and recruitment is time intensive during a period when the pandemic seems to be changing often. Delays can impact the gathering of much needed data. Another consideration with prospective cohort studies is participant retention. To bolster retention, we send email reminder notifications for survey completion. Future strategies to promote retention include providing incentives to enroll and for completing surveys.

One component of success for this study hinges on the setup and active monitoring of the REDCap database. The REDCap workflow was constructed to ensure seamless flow from consent and assent to the baseline survey. Initially, participants had the option to select a preferred communication method: parent communication and youth communication. Through active data monitoring we discovered a lower participation rate (nearly half) among those that preferred youth communication. The communication method was changed to parent communication only. For each adolescent that enrolls, the parent/guardian receives an email with a unique personalized link for each adolescent.

## Conclusion

Our study describes a longitudinal investigation of COVID-19, depressive symptoms, and anxiety symptoms. It is unique in that it applies Intersectionality Theory as a guiding framework. Results are expected to inform future practice, policy, and research.

## Data availability statement

The original contributions presented in the study are included in the article/supplementary material, further inquiries can be directed to the corresponding author.

## Author contributions

Conceptualization: VL, PG-F, KP-B, MJ, OD, MD, DC, and LB. Methodology: FC-M, VL, PG-F, MJ, OD, MD, DC, and LB. Analysis: NM and PG-F. Writing: VL, NM, OD, MD, KP-T, and PG-F. Supervision: VL and PG-F. All authors contributed to the article and approved the submitted version.
